# Amygdala subnuclei volumes and anxiety behaviors in children and adolescents with autism spectrum disorder, attention deficit hyperactivity disorder, and obsessive–compulsive disorder

**DOI:** 10.1002/hbm.26005

**Published:** 2022-07-12

**Authors:** Diane Seguin, Sara Pac, Jianan Wang, Rob Nicolson, Julio Martinez‐Trujillo, Evdokia Anagnostou, Jason P. Lerch, Christopher Hammill, Russell Schachar, Jennifer Crosbie, Elizabeth Kelley, Muhammad Ayub, Jessica Brian, Xudong Liu, Paul D. Arnold, Stelios Georgiades, Emma G. Duerden

**Affiliations:** ^1^ Physiology & Pharmacology, Schulich School of Medicine and Dentistry Western University London Canada; ^2^ Neuroscience, Schulich School of Medicine and Dentistry Western University London Canada; ^3^ Biomedical Engineering, Faculty of Engineering Western University London Canada; ^4^ Psychiatry, Schulich School of Medicine and Dentistry University of Western Ontario London Canada; ^5^ Bloorview Research Institute, Holland Bloorview Kids Rehabilitation Hospital University of Toronto Toronto Canada; ^6^ The Hospital for Sick Children Toronto Canada; ^7^ Wellcome Centre for Integrative Neuroimaging University of Oxford, FMRIB, Nuffield Department of Clinical Neurosciences Oxford UK; ^8^ Department of Medical Biophysics University of Toronto Toronto Canada; ^9^ Department of Psychology Queen's University Kingston Canada; ^10^ Department of Psychiatry Queen's University Kingston Canada; ^11^ Queen's Genomics Lab at Ongwanada (QGLO) Ongwanada Resource Center Kingston Canada; ^12^ Department of Psychiatry Cumming School of Medicine University of Calgary Calgary Canada; ^13^ Department of Medical Genetics, Cumming School of Medicine University of Calgary Calgary Canada; ^14^ Department of Psychiatry and Behavioural Neurosciences McMaster University Hamilton Canada; ^15^ Applied Psychology, Faculty of Education Western University London Canada

**Keywords:** autism spectrum disorder, amygdala subnuclei, anxiety, attention deficit hyperactivity disorder, obsessive compulsive disorder

## Abstract

Alterations in the structural maturation of the amygdala subnuclei volumes are associated with anxiety behaviors in adults and children with neurodevelopmental and associated disorders. This study investigated the relationship between amygdala subnuclei volumes and anxiety in 233 children and adolescents (mean age = 11.02 years; standard deviation = 3.17) with autism spectrum disorder (ASD), attention deficit hyperactivity disorder (ADHD), and children with obsessive compulsive disorder (OCD), as well as typically developing (TD) children. Parents completed the Child Behavior Checklist (CBCL), and the children underwent structural MRI at 3 T. FreeSurfer software was used to automatically segment the amygdala subnuclei. A general linear model revealed that children and adolescents with ASD, ADHD, and OCD had higher anxiety scores compared to TD children (*p* < .001). A subsequent interaction analysis revealed that children with ASD (B = 0.09, *p* < .0001) and children with OCD (B = 0.1, *p* < .0001) who had high anxiety had larger right central nuclei volumes compared with TD children. Similar results were obtained for the right anterior amygdaloid area. Amygdala subnuclei volumes may be key to identifying children with neurodevelopmental disorders or those with OCD who are at high risk for anxiety. Findings may inform the development of targeted behavioral interventions to address anxiety behaviors and to assess the downstream effects of such interventions.

## Graphical abstract

This study investigated the relationship between amygdala subnuclear volumes and anxiety in 233 children (mean age = 11 years; standard deviation = 3.17) with autism spectrum disorder (ASD), attention deficit hyperactivity disorder (ADHD) and children with obsessive compulsive disorder (OCD), as well as typically developing (TD) children. A general linear model revealed that children with ASD, ADHD, OCD had higher anxiety scores compared to TD children (*p* < .001) and a subsequent interaction analysis revealed that children with ASD (B = 0.09, *p* < .0001) and children with OCD (B = 0.1, *p* < .0001) with high anxiety had larger right central nuclei volumes compared with TD children. Amygdala subnuclei volumes may be key to identifying children with neurodevelopmental disorders at high risk for anxiety.

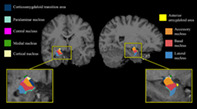

## 1 INTRODUCTION

Neurodevelopmental disorders (NDD) are classified as a group of disorders with onsets prior to adulthood. These conditions may produce challenges with social, academic, personal, or occupational functioning (American Psychiatric Association, 2013). NDDs associated with early alterations in brain development include autism spectrum disorder (ASD) and attention deficit hyperactivity disorder (ADHD). Children with NDDs are more likely than their typically developing (TD) peers to report symptoms of anxiety. Additionally, children with obsessive compulsive disorder (OCD) also report high levels of anxiety related to fears of catastrophic events, death, or illness, in relation to their developmental level and needs. Anxiety is an emotional reaction to stressful situations or situations where the outcome is uncertain. Anxiety can be an adaptive mechanism for coping in these situations.

The increased prevalence of anxiety in children and adolescents with NDDs and OCD compared with TD children remains high. For instance, over 85% of children with ASD display symptoms and signs of anxiety, with nearly 40% of children having a recognized anxiety disorder (van Steensel & Heeman, 2017). The prevalence of anxiety and depression are similarly high in individuals with ADHD, with up to 50% having a comorbid anxiety disorder (Ivarsson et al., 2008). Previously categorized as an anxiety disorder in the DSM‐IV, based on the DSM‐5 criteria OCD is characterized by intrusive thoughts or obsessions, with high levels of anxiety seen in diagnosed children, and approximately 40% meeting criteria for an anxiety disorder (Kessler et al., 2006). The prevalence of anxiety in ASD and ADHD is higher than that seen in TD children where only a quarter of the population has symptoms of anxiety (Costello et al., 2005). Comorbidities such as anxiety may exacerbate the symptoms of NDDs, including core symptomatology in ASD such as difficulties with social interaction and reduced eye contact, as well as impulsivity and hyperactivity behaviors characteristic of ADHD.

Anxiety is a major risk factor for adverse mental health outcomes and is associated with reduced quality of life (Olatunji et al., 2007; Parker et al., 1999). Moreover, social anxiety can impact adherence to treatment for anxiety disorders (Geraets et al., 2019), which involve engaging in social interaction with a therapist and in many settings are offered in a group context. While anxiety disorders remain a significant concern in children with NDDs and OCD, few risk factors or objective biomarkers have been identified.

A key feature of anxiety disorders is difficulty with regulating negative emotions. The amygdala is central to affective processing and is a major component of the limbic system and affective loop of the cortico‐striato‐thalamo‐cortical circuit (Alexander et al., 1986). In this circuit, the amygdala receives highly processed somatosensory, visual, auditory, and visceral inputs. It contains at least 13 subnuclei, with the basal, lateral, and accessory basal nuclei forming the basolateral amygdala (BLA) complex, which connects sensory stimuli to brain regions involved in higher order social cognition. The BLA also has reciprocal connections with the orbitofrontal cortex, anterior cingulate cortex (ACC), and the medial prefrontal cortex (mPFC) (Adolphs, 2010). The BLA is central to anxiety processing as it integrates information from cortical and thalamic sensory inputs to generate fear and anxiety‐related behaviors (Felix‐Ortiz & Tye, 2014). The central nucleus (Ce) is the main output nucleus of the amygdala and mediates aspects of fear and anxiety processing, as Ce neurons project to the hypothalamus, basal forebrain, and brainstem sites involved in different aspects of stress responses (Amaral et al., 1992).

Altered amygdala development is also a key feature of the neuroanatomy of children with NDDs (Schumann et al., 2004, 2011) particularly children with ASD (Dalton et al., 2005; Mosconi et al., 2009). Previous studies have also demonstrated that larger amygdala volumes predict anxiety symptoms in children with ASD (Juranek et al., 2006). In a longitudinal study using volumetric MRI to examine the development of the amygdala in children with ASD compared to TD children, it was demonstrated that alterations in the development of this structure were associated with the clinical presentation of direct eye contact (Barnea‐Goraly et al., 2014). Children with ASD with co‐occurring anxiety have decreased right amygdala volumes relative to TD children as well as compared to children with ASD without anxiety (Herrington et al., 2017), suggesting the neurodevelopmental trajectory is distinct in youth with ASD and co‐occurring anxiety. Another recent study reported no association among anxiety behaviors and whole amygdala volumes (Yarger et al., 2021). Functional neuroimaging studies in children with OCD have also noted hyperactivation in the amygdala in response to aversive/stressful stimuli (Via et al., 2014). Abnormal connectivity between the prefrontal cortex and amygdala in an ADHD group with a significantly reduced bilateral size over the BLA complex also suggest limbic system involvement in the pathophysiology of ADHD that may contribute to behavioral disinhibition (Plessen et al., 2006).

Pediatric anxiety disorder studies have reported atypical structural and functional development of the amygdala. Children, adolescents, and adults with anxiety disorders demonstrated larger amygdala volumes (de Bellis et al., 2000; Machado‐de‐Sousa et al., 2014). In particular, the right amygdala volumes were larger in children with anxiety disorder (de Bellis et al., 2000). Heightened functional activity in the amygdala in response to emotional faces in children with anxiety has been reported as well as in adults with social anxiety (Fonzo et al., 2015; Thomas et al., 2001). The mechanisms underlying these relationships are unclear. Potentially, anxiety‐induced hyperexcitability in the amygdala may be associated with subsequent cellular dysmaturation and resulting in smaller amygdala volumes (Blackmon et al., 2011). However, another study that reported that enlarged amygdala subnuclei volumes predicted higher levels of childhood anxiety interpreted the findings to result from increased synaptic formation and decreased pruning, which may be related to extended exposure to stress hormones (Qin et al., 2014). Abnormalities in white matter microstructure within the amygdala and its connections to nearby structures correlate highly with scores on anxiety questionnaires in children and youths with generalized anxiety disorders (Jalbrzikowski et al., 2017).

Given the known role of the amygdala in anxiety, we wanted to determine whether alterations in the volumes of the subnuclei of the amygdala were associated with anxiety behaviors in a large population of boys and girls with ASD, ADHD, OCD, as well as TD children and adolescents. We hypothesized that high levels of anxiety, irrespective of diagnosis, would be predicted by enlarged amygdala subnuclei volumes. Anxiety was assessed using the Child Behavior Checklist (CBCL), in line with previous work (Juranek et al., 2006; Qin et al., 2014). The CBCL is a standardized parent‐report questionnaire with strong reliability and validity (Achenbach, 2001). Our first aim was to examine anxiety scores in children with NDD, OCD, and TD children. Our second aim was to examine the amygdala subnuclei volumes in relation to anxiety scores, adjusting for demographic variables. We hypothesized that children with NDDs and OCD will exhibit higher anxiety and that these children with higher anxiety will have altered amygdala nuclei volumes compared to TD children.

## 2 METHODS

### 2.1 Participants

Children with NDDs (ASD, ADHD), OCD, and TD children were recruited to participate in the Province of Ontario Neurodevelopmental Disorders Network (POND) study (pond-network.ca). POND recruitment sites included five sites across Ontario, Canada and included Holland Bloorview Kids Rehabilitation Hospital and The Hospital for Sick Children, Toronto, McMaster Children's Hospital, Hamilton, Queen's University, Kingston, and Lawson Health Research Institute, London. Inclusion criteria were age less than 21 years, and a clinical diagnosis of ADHD, ASD, or OCD or TD children and youth. The POND study began data collection in 2012 at which time OCD was included in the DSM‐IV as an anxiety disorder. Although OCD is now categorized in the DSM‐5 (1) as its own class of disorders, it remains a disorder associated with altered brain development and onset during childhood and the POND study continues to recruit children with an OCD diagnosis. Participant inclusion has been described elsewhere (Baribeau et al., 2019; Kushki et al., 2019). Briefly, participants were recruited into the study based on the primary diagnosis. Comorbid symptomatology was recorded using symptom surveys in participants. Standardized research assessments were used to confirm the primary clinical diagnosis in participants. The Autism Diagnostic Observation Schedule—2 (ADOS‐2) (Lord et al., 2000), which involves live behavioral assessment of the child in semistructured activities by a trained clinician, and the Autism Diagnostic Interview‐Revised (ADI‐R) (Lord et al., 1994), a parent report of their child's development and behaviors, were used to confirm a primary diagnosis of ASD. The Kiddie‐Schedule for Affective Disorders and Schizophrenia (K‐SADS) (Kaufman et al., 1997), a semi‐structured parent interview, and the Children's Yale‐Brown Obsessive–Compulsive Scale (Scahill et al., 1997), which obtains behavioral information from both the child and parent, were used to confirm an OCD diagnosis. ADHD diagnoses were confirmed with use of the Parent Interview for Child Symptoms (Ickowicz et al., 2006). Clinical judgment and standardized tests were used. The data for standardized tests were not available for all participants. TD children without a diagnosis of a NDD were recruited through the community (e.g., advertising in public transit, hospitals, social media). Exclusion criteria included a diagnosis of a neurodevelopmental or psychiatric disorder, or a first‐degree relative with a diagnosis of a NDD. This study was conducted in accordance with the Declaration of Helsinki and the research ethics boards at each of the five sites (Holland Bloorview Kids Rehabilitation Hospital, The Hospital for Sick Children, McMaster Children's Hospital, Queen's University, and Western University [Lawson] research ethics boards) approved the study. Parents provided informed consent and children provided assent.

## 3 ANXIETY ASSESSMENT

Parents completed the CBCL for children aged 6–18 years, which is an age‐appropriate standardized parent‐report questionnaire. The anxiety (CBCL‐A) subscale from the CBCL (Achenbach, 2001) was used to determine anxiety problems. Parents are asked about behaviors pertaining to the current time period or up to 6 months before the assessment.

The 16‐item CBCL‐A includes 6 items from the CBCL attention problem subscale (CBCL‐AP) and 10 additional items regarding somatic symptoms. The CBCL‐A has demonstrated high internal consistency (Kendall et al., 2007).

## 4 MAGNETIC RESONANCE IMAGING

All brain imaging for the present study was performed on the same 3 T MRI system (Tim Trio/upgraded to Prisma Fit, Siemens Healthcare, Germany) at the Hospital for Sick Children in Toronto, Ontario. Anatomical scans were acquired using a three‐dimensional T1‐weighted MPRAGE sequences (Tim Trio: 12‐channel head coil, field of view[FOV] = 192 × 240 × 256 mm, 1 × 1 × 1mm voxels, repetition time [TR] = 2300, inversion time [TI] = 900 ms, echo time[TE] = 2.96 s, flip angle = 9°, GRAPPA = 2; Prisma fit: 20‐channel head coil, FOV = 240 × 300 × 320 mm, 0.8 × 0.8 × 0.8 mm voxels, TR = 1870, TI = 945, TE = 3.14 s, flip angle = 9°, GRAPPA = 2).

### 4.1 Amygdala segmentation

The entire amygdala and several of its subnuclei were segmented automatically using FreeSurfer (http://surfer.nmr.mgh.harvard.edu) version 6.0. The algorithm is based on Bayesian inference, and the amygdala atlas was developed using ex vivo human prosections (*n* = 10). The prosections were scanned using ultra‐high field MRI. T1‐weighted structural images were acquired with sub‐millimetric resolution of 0.1 mm. The nuclei were manually labeled by an expert rater to create an atlas of the amygdala nuclei (Saygin et al., 2017). In a previous validation study, the atlas was applied in an automatic segmentation pipeline using two publicly available datasets acquired at 3 T MRI in patient and TD populations (Saygin et al., 2017).

**FIGURE 1:**
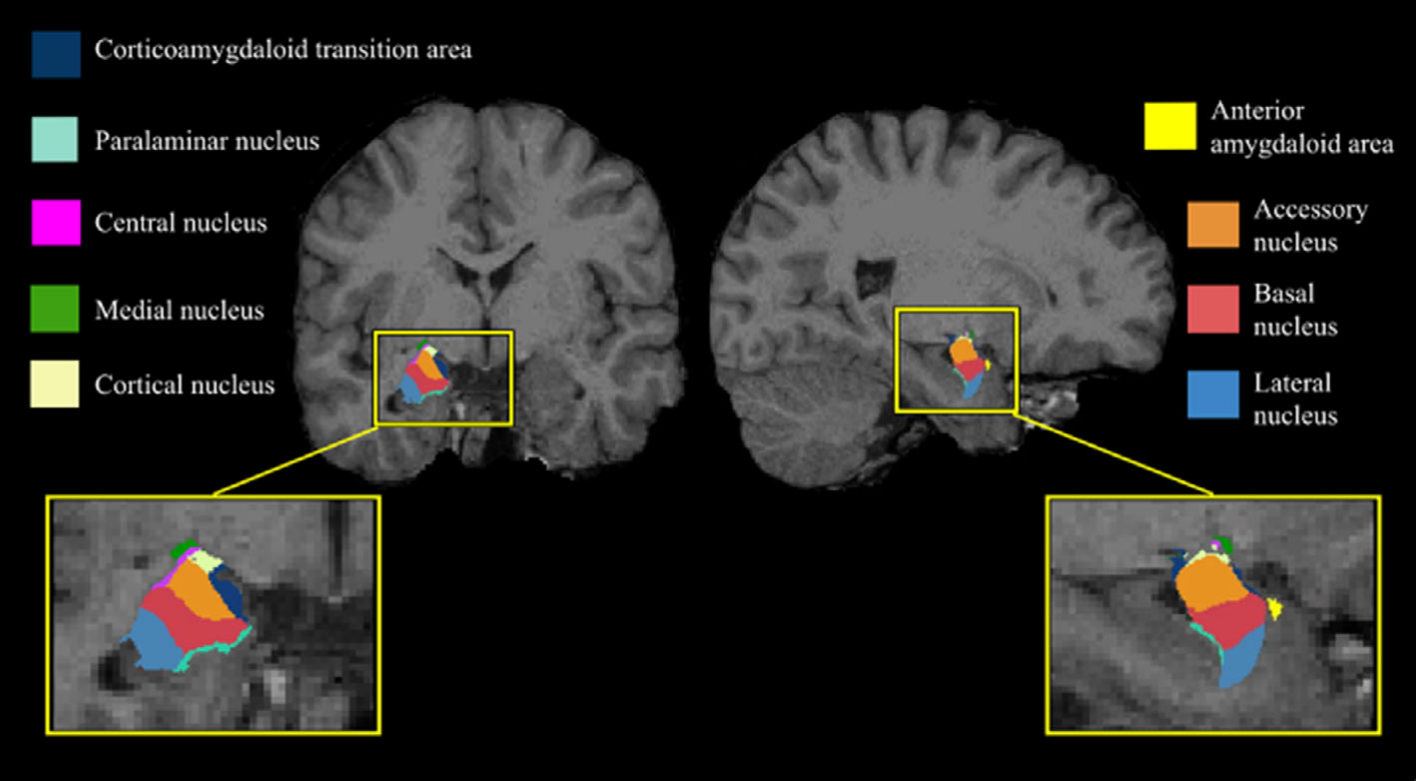
Amygdala atlas registered to a representative T1‐weighted image obtained in a healthy control participant. CTA, corticoamygdaloid transition area

In the current study, the amygdala was segmented into the Ce, lateral, basal, accessory basal, cortical, medial, and paralaminar nuclei as well as the corticoamygdaloid transition area (CTA) and the anterior amygdaloid area (AAA, Figure 1). Postprocessed images were visually inspected to determine the accuracy of the algorithm used to segment the nuclei. The nuclei forming the BLA complex (basal, lateral, and accessory basal nuclei) were summed and were used as predictor variables in the subsequent analyses.

### 4.2 Statistical analysis

Statistical analyses were carried out using SPSS version 27 (Statistical Package for the Social Sciences, IBM, Chicago, IL). To address our first aim, the raw CBCL‐A scores were assessed in relation to diagnosis group using a generalize linear model (GLM). The raw CBCL‐A scores were the dependent variable and diagnosis group was an independent variable, adjusting for sex and age. Given that we had one a priori hypothesis, the alpha level for the statistical model to address this aim was set at *p* = .05. To address our second aim, in two separate GLMs, one for the right amygdala subnuclei volumes and the other for the left amygdala subnuclei volumes (Ce, BLA, cortical, medial and paralaminar nuclei, and the CTA and AAA) the association with anxiety was assessed. The subnuclei volumes were the independent variables and the CBCL‐A scores were the dependent variables. Diagnostic group (ASD, ADHD, OCD, TD) was a factor in these models. Both models were adjusted for sex, age, and total cerebral volumes. As we had two a priori hypotheses for our second aim regarding the association of diagnosis and left and right amygdala subnuclei volumes on anxiety scores, the alpha level for the statistical tests was set at *p* = .025. In order to further assess the patterns of subnuclei volumes in relation to anxiety scores we conducted a k‐means cluster analysis. The CBCL‐A scores and the volumes of the subnuclei were transformed to z scores. Separate k‐means cluster analyses were conducted for the subnuclei in relation to anxiety scores. As the k‐means cluster analyses were exploratory, the alpha level was set at *p* = .05.

## 5 RESULTS

A total of 301 children participated in the study, 124 (41%) of whom had a diagnosis of ASD, 69 (23%) had a diagnosis of ADHD, 45 (15%) had a diagnosis of OCD and 63 (21%) were TD children. The children's ages ranged from 4 to 18 years (10.72 ± 4.29 years). The majority of the children were males 221 (73%) in the total sample as well as in the subgroups of children (*X* ^2^ = 14.5, *p* = .002). All participants included in the study were recruited between February 2012 and December 2018.

### 5.1 Anxiety assessments

Parents of 233 (77%) children aged 6 years and older enrolled in the study completed the CBCL‐A (one child was nearly 6 years old). OThe CBCL‐A was completed in 43% (*n* = 101) of children with ASD, 25% (*n* = 59) of children with ADHD, 15% (*n* = 36) in children with OCD, and 16% (*n* = 37) of TD children (Table 1). The raw anxiety scores ranged from 0 to 12 (overall mean: 3.64) and significantly differed by diagnostic group (*p* < .001). Children with ASD (mean = 4.62, range = 0–12) and OCD (mean = 5.17, range = 0–12) had the highest anxiety scores.

**TABLE 1:** Participant characteristics

	Total	TD	ASD	ADHD	OCD	*p* value
*n* (%)	233	37 (16)	101 (43)	59 (25)	36 (15)	
Males (%)	176 (76)	25 (68)	77 (76)	50 (85)	24 (67)	.002
Age [SD]	11.02 [3.17]	10.78 [3.5]	11.46 [3.54]	10.07 [2.46]	11.46 [3.54]	.04
*n* Age ranges						
5–6 years	13	2	7	4	0	‐
7–11 years	98	17	34	32	15	‐
12–15 years	99	14	44	21	20	‐
>16 years	23	4	16	2	1	‐
Children's Behavior Checklist						
Total anxiety [SD]	3.64 [3.11]	0.78 [1.27]	4.62 [3.10]	2.83 [2.49]	5.17 [3.09]	<.001

*Note*: Values for age and raw anxiety scores from the Children's Behavior Checklist are provided as means. Probability values provide results using analysis of covariance for continuous measures and Chi‐square tests for categorical measures comparing the data from the four groups of children.

Abbreviations: ADHD, attention deficit hyperactivity disorder; ASD, autism spectrum disorder; OCD, obsessive compulsive disorder; SD, standard deviation; TD, typically developing.

### 5.2 Amygdala volumes

Total brain volumes and the amygdala subnuclei were extracted from the T1‐weighted images of the 233 children for whom anxiety assessments were available. No effects of scanner upgrade were evident in the amygdala subnuclei volumes (*F* = 1.40, *p* = .12).

We examined whether the right and left subnuclei volumes differed by diagnosis type in two separate multivariate general linear models, adjusting for sex, age, and total cerebral volumes. The right AAA volumes were significantly larger in children and adolescents with OCD compared to those with ASD and who were TD (*p* = .02). Additionally, the paralaminar nucleus was larger in the OCD group compared with the TD group (*p* = .001). Both models were Bonferroni corrected for multiple comparisons. No differences in the left hemisphere subnuclei volumes were seen among the diagnostic groups (*p* > .05).

### 5.3 Anxiety scores are predicted by diagnosis and amygdala volumes

**FIGURE 2:**
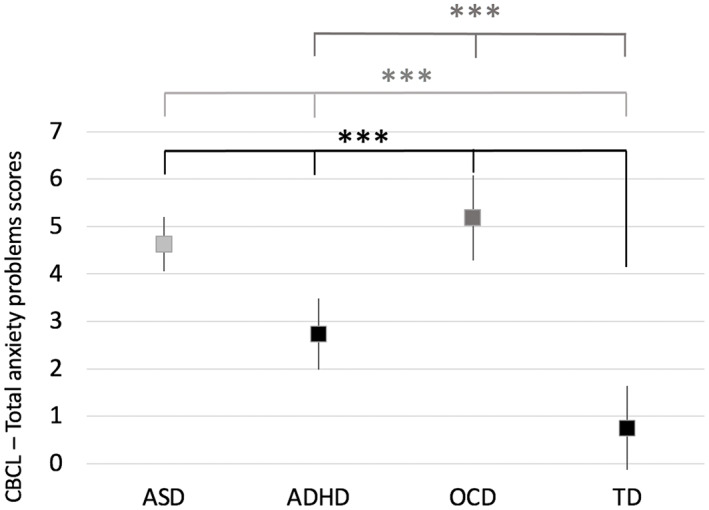
Mean total anxiety scores on the CBCL (*y* axis) differed significantly based on diagnosis in children with autism spectrum disorder (ASD), attention deficit hyperactivity disorder (ADHD), obsessive compulsive disorder (OCD), and typically developing (TD) children (*p* < .001, black). A pairwise analysis revealed that the ASD group had significantly higher anxiety scores than the ADHD and TD groups (both, *p* < .001, light grey). Additionally, the OCD group had higher anxiety scores relative to the ADHD and the TD groups (both, *p* < .001, dark grey). Error bars represent 95% confidence intervals. ***p < .001

The CBCL‐A scores were examined in relation right amygdala subnuclei volumes. Diagnostic group showed a significant main effect (*p* < .001) for anxiety scores. Children with ASD (B = 3.8, *p* < .001), ADHD (B = 1.8, *p* = .002), and OCD (B = 4.1, *p* < .001) had higher anxiety scores relative to TD children (Figure 2. Table 2). A pairwise analysis revealed that the ASD group had significantly higher anxiety scores than the ADHD and TD groups (both, *p* < .001). Additionally, the OCD group had higher anxiety scores relative to the ADHD and the TD groups (both, *p* < .001). The pairwise results were Bonferroni corrected for multiple comparisons.

**TABLE 2:** Results of a generalized linear model examining the total anxiety scores in relation to right amygdala subnuclei volumes

		95% CI		
	B	Lower	Upper	*p* value
ASD	3.8	2.99	5.28	<.001
ADHD	1.8	0.98	3.67	.002
OCD	4.1	3.13	6.13	<.001
TD (reference)	0	‐	‐	‐
Males	0.1	−1.25	0.71	.9
Age	−0.1	−0.26	0.01	.2
Amygdala nuclei				
Central nuclei	0.07	0.01	0.1	.02
Basolateral nuclei	−0.01	−0.01	0.001	.09
Anterior amygdaloid area	0.07	0.01	0.1	.02
Medial nuclei	0.01	−0.1	0.1	.8
Cortical nuclei	0.01	−0.1	0.2	.9
Cortex‐amygdala transition zone	0.0001	−0.03	0.03	.99
Paralaminar nucleus	0.1	−0.1	0.2	.3
Total cerebral volume	−0.000001	−0.00001	0.000004	.9

*Note*: Results of a generalized linear model examining the association of total anxiety scores in relation to diagnostic group and amygdala volumes, adjusting for sex, age, and total cerebral volumes.

Abbreviations: ASD, autism spectrum disorder; ADHD, attention deficit hyperactivity disorder; IQR, interquartile range; OCD, obsessive compulsive disorder; TD, typically developing.

Larger right Ce nuclei volumes predicted total scores on the CBCL‐A (B = 0.07, *p* = .02), adjusting for sex, age, and total cerebral volumes. In the same model, larger AAA predicted higher CBCL‐A scores (B = 0.07, *p* = .03, Table 2), adjusting for the same confounders. Neither age nor sex predicted anxiety scores (both, *p* > .05).

A subsequent interaction analysis to examine the association of diagnostic group and right Ce volumes with CBCL‐A scores indicated that children and adolescents with ASD (B = 0.09, *p* < .001, Table 3) and children and adolescents with OCD (B = 0.1, *p* < .001) had larger volumes and higher anxiety in comparison to TD children, adjusting for sex, age and total cerebral volumes. Similar results were found for diagnostic group and right AAA volumes and the association with anxiety scores was evident, whereby higher anxiety scores were associated with larger volumes in children and adolescents with ASD (B = 0.06, *p* = .02) and OCD (B = 0.06, *p* = .01), adjusting for sex, age, and total cerebral volume.

**TABLE 3:** Results of a generalized linear model of total anxiety scores: Interaction analysis for diagnosis group and right central nucleus volumes

		95% CI		
	B	Lower	Upper	*p* value
Males	0.1	−0.8	0.98	.8
Age	−0.1	−0.2	0.03	.1
ASD * central nucleus	0.09	0.04	0.1	<.0001
ADHD * central nucleus	0.05	0.001	0.1	.05
OCD * central nucleus	0.1table	0.05	0.2	<.0001
TD * central nucleus	0.02	−0.04	0.07	.6
Total cerebral volume	−0.000001	−0.000004	0.000003	.8

*Note*: Results of a generalized linear model examining the association of total anxiety scores in relation to diagnostic group and right central volumes in an interaction analysis, adjusting for site, sex, age, and total cerebral volumes.

Abbreviations: ASD, autism spectrum disorder; ADHD, attention deficit hyperactivity disorder; IQR, interquartile range; OCD, obsessive compulsive disorder; TD, typically developing.

No association among anxiety scores, diagnostic group and left amygdalar subnuclei volumes were evident (all, *p* > .09).

**FIGURE 3:**
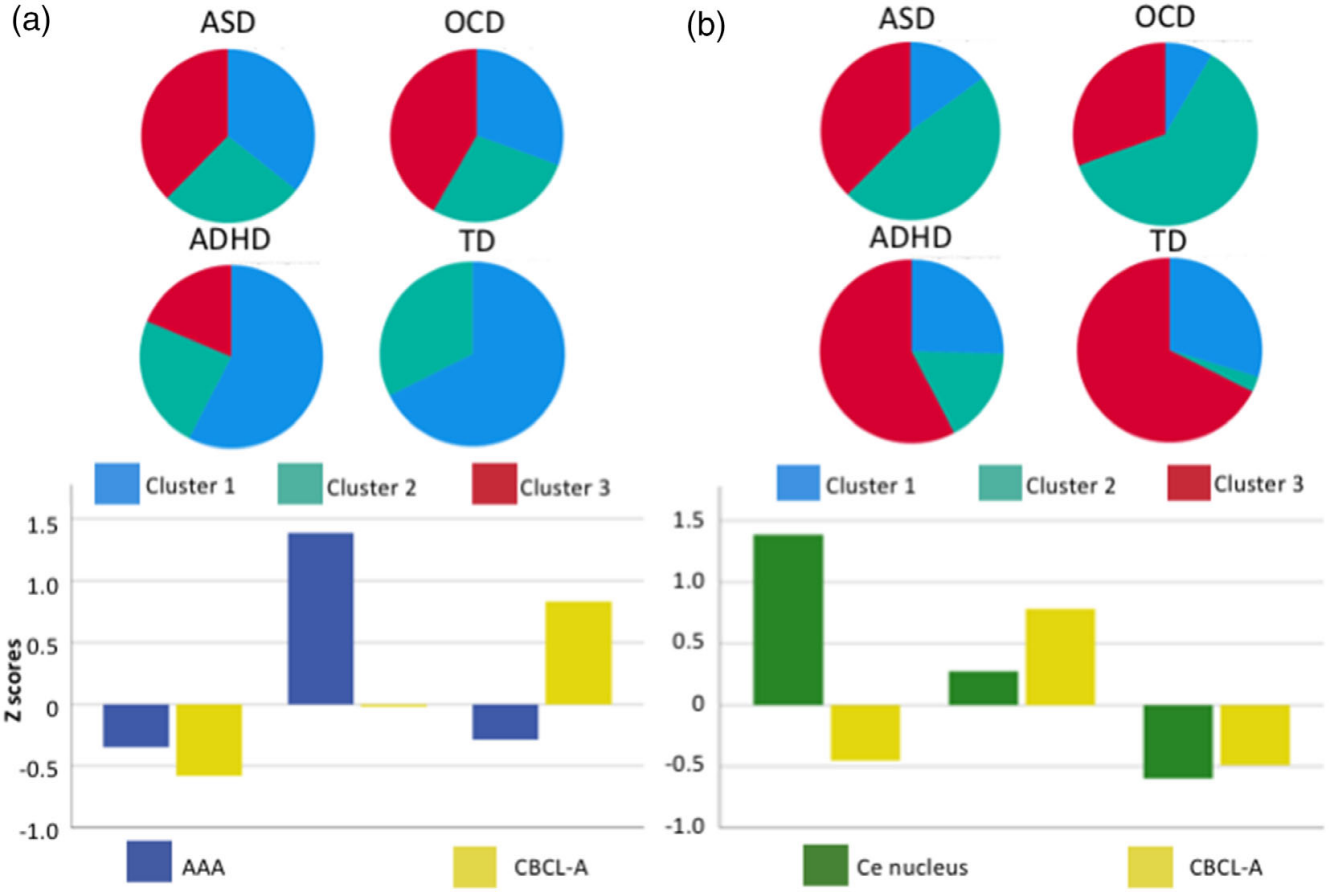
(a) Results of a k‐means three‐cluster model applied to the volume data for the anterior amygdaloid area (AAA) and the CBCL‐A scores (bottom) and cluster membership among the diagnostic groups (top). (b) Results of a three‐cluster model for the volume data obtained from the Ce (central) nucleus and CBCL‐A scores (bottom) and cluster membership among the diagnostic groups (top). ASD, autism spectrum disorder; ADHD, attention deficit hyperactivity disorder; OCD, obsessive compulsive disorder; TD, typically developing

K‐means cluster analyses were implemented to evaluate whether diagnostic groups would demonstrate differences in amygdala subnuclei volumes relative to anxiety behaviors. Subsequent one‐way ANOVAs with Bonferroni post‐hoc analyses were run to examine the differences between diagnostic groups and cluster membership. The CBCL‐A, volumes of the AAA and Ce nuclei were transformed to z scores. A cluster analysis for the AAA volumes and anxiety scores was run followed by a subsequent cluster analysis to examine the Ce volumes and anxiety scores. Both cluster analyses revealed three distinct clusters (*p* < .01). The follow up ANOVA examining CBLC‐A scores and AAA subnuclei volumes revealed both CBCL‐A (*F*(2,231) = 164.71, *p* < .01) and AAA volumes (*F* (2,231) = 201.71, *p* < .01) significantly differed between clusters. Cluster 1 comprised low CBCL‐A scores and smaller AAA subnuclei volumes. Cluster 2 included low‐to‐moderate CBCL‐A scores and large AAA subnuclei volumes while Cluster 3 includes high CBCL scores and small AAA volumes (Figure 3a). A Bonferroni post hoc test revealed CBCL‐A scores significantly differed between all three clusters (*p* < .01). For AAA volumes, Clusters 1 and 3 (*p* < .01) and Clusters 2 and 3 (*p* < .01) differed in volume, while Clusters 1 and 2 did not display significantly different volumes. The majority of ADHD and TD participants belong to Cluster 1 (Table 4). Cluster 3 was entirely comprised of children with a diagnosis. For the second cluster analysis examining the Ce nucleus and the CBCL‐A scores, both CBCL‐A scores (*F*(2,231) = 189.23, *p* < .001) and Ce subnuclei volumes (*F*(2,231) = 162.13, *p* < .01) differed across the three clusters. Cluster 1 is comprised of large Ce volumes and low CBCL‐A scores. Cluster 2 is comprised of moderate Ce volumes and high CBCL‐A scores while Cluster 3 includes small Ce volumes and low CBCL‐A scores (Figure 3b). Bonferroni post hoc revealed Ce volumes significantly differed among all cluster comparisons (*p* < .01). CBCL‐A scores significantly different between Clusters 1 and 2 and between Clusters 2 and 3 (*p* < .01) but did not significantly differ between Clusters 1 and 3 (*p* > .05), which displayed similar CBCL‐A scores (Table 5). The ASD and OCD groups showed similar clustering patterns, with over 50% belonging to Cluster 2 and were significantly different from the clustering patterns displayed by the ADHD (*p* < .001) and TD (*p* < .001) groups, in which over 50% of participants were included in Cluster 3. All analyses were Bonferroni corrected for multiple comparisons.

**TABLE 4:** CBCL‐A and AAA volumes

	TD	ASD	ADHD	OCD	Total
	*n* (%)	*n* (%)	*n* (%)	*n* (%)	*n*
Cluster 1	25 (23.4)	36 (33.6)	34 (31.8)	11 (10.3)	106
Cluster 2	12 (19.0)	27 (42.9)	14 (22.2)	10 (15.9)	63
Cluster 3	0 (0.00)	38 (59.4)	11 (17.2)	15 (23.4)	64
Total	37	101	59	36	233

*Note*: Results of a k‐means cluster analysis with a three‐cluster model separated by diagnostic group.

Abbreviations: ASD, autism spectrum disorder; ADHD, attention deficit hyperactivity disorder; IQR, interquartile range; OCD, obsessive compulsive disorder; TD, typically developing.

**TABLE 5:** CBCL‐A and Ce nuclei volumes: Three cluster model membership

	TD	ASD	ADHD	OCD	Total
	*n* (%)	*n* (%)	*n* (%)	*n* (%)	*n*
Cluster 1	11 (24.4)	15 (33.3)	15 (33.3)	3 (6.7)	44
Cluster 2	1 (1.2)	48 (59.3)	10 (12.3)	22 (27.2)	81
Cluster 3	25 (23.1)	38 (35.2)	34 (31.5)	11 (10.2)	108
Total	37	101	59	36	233

*Note*: Results of a k‐means cluster analysis with a three‐cluster model separated by diagnostic group.

Abbreviations: ASD, autism spectrum disorder; ADHD, attention deficit hyperactivity disorder; IQR, interquartile range; OCD, obsessive compulsive disorder; TD, typically developing.

## 6 DISCUSSION

In a multisite cohort study of children and adolescents with NDDs and OCD using standardized anxiety measures and structural neuroimaging, we report that children with ASD, ADHD, and OCD had higher anxiety scores relative to TD children and adolescents, based on parent report. Through using an automatic segmentation algorithm of the amygdala subnuclei, we report macrostructural differences in relation to anxiety scores. Larger volumes of the right Ce nuclei, the main output nucleus of the amygdala, a sensory input nuclear complex, predicted higher anxiety scores. Larger volumes of the right Ce nucleus and AAA predicted higher anxiety scores in children with ASD and OCD, a relationship not seen in TD children and adolescents. Additionally, larger volumes of the AAA were also associated with higher anxiety scores in children and adolescents with ASD and those with OCD. No association between amygdala subnuclei volumes, ADHD diagnosis and total anxiety scores was evident. TD children and adolescents did not demonstrate an association between Ce volumes and anxiety. Regionally specific alterations in amygdala subnuclei morphology may represent an important brain–behavior relationship for anxiety behaviors and related affective disorders for children and adolescents with ASD and OCD.

### 6.1 Anxiety in children with neurodevelopmental disorders and obsessive compulsive disorder

In a large group of children and adolescents with ASD and OCD, both groups had greater anxiety compared to TD children and adolescents. Our findings are in agreement with previous reports indicating that anxiety disorders are common in children and adolescents with ASD (Bitsika et al., 2016; Wijnhoven et al., 2019). Additionally, children and adolescents with ADHD and children with OCD are also at risk for the development of generalized anxiety disorder (Osland et al., 2018).

The greater risk for anxiety behaviors in children and adolescents with ASD and OCD is likely influenced by clinical phenotypes. For example, social cognition difficulties in children and adolescents with ASD may lead to heightened anxiety in social situations (Cooper et al., 2014; Jansen et al., 2020; Pepper et al., 2018). Additionally, children and adolescents with ASD (Duerden et al., 2012) and OCD can also engage in repetitive behaviors including rituals, sameness behaviors and insistence on routines, which some children and adolescents may exhibit in order to manage anxiety. Social‐cognitive difficulties have been associated with alterations in amygdala volumes in TD children and adolescents (Evans et al., 2015). Social cognition and repetitive behaviors were not assessed in all children and adolescents in the current study. A future avenue of research would be to examine the association of social‐communication difficulties and repetitive behaviors with amygdala volumes in TD children and children with ASD, ADHD, and OCD.

### 6.2 Circuitry of the amygdala subnuclei and anxiety behaviors

In the current work through use of an advanced automatic segmentation algorithm, we examined the volumes of the subnuclei of the amygdala in relation to anxiety behaviors. Children with OCD had larger right AAA and paralaminar volumes compared to TD children. Additionally, AAA volumes were enlarged in the OCD group relative to children with ASD. Altered amygdala subnuclei volumes in adults with OCD have been reported previously (Zhang et al., 2020) and were interpreted to relate to cognitive and affective deficits seen in OCD; however, previous findings were not related to anxiety behaviors in this population.

Our results indicate enlarged volumes of the Ce and AAA volumes were associated with anxiety behaviors in children and adolescents with ASD and OCD. A cluster analyses revealed comparable associations between amygdala subnuclei volumes and CBCL‐A scores between children and adolescents with ASD and OCD, suggesting shared neurophysiological and behavioral correlates of anxiety. Previous research with children with generalized anxiety disorder also reported increased amygdala volumes lateralized to the right hemisphere (de Bellis et al., 2000). Our results demonstrating regional increases in amygdala volumes, particularly in the Ce nucleus and the AAA, and the association with anxiety in children and adolescents with ASD and OCD may underlie some of the variation reported in previous studies that examined whole amygdala volumes relative to anxiety.

Reports of both larger and smaller volumes of the whole amygdala in relation to anxiety have been reported in children with ASD (Juranek et al., 2006). Smaller amygdala volumes have been reported in adults with OCD (Atmaca et al., 2008; Gurok et al., 2019); however, the association with co‐morbid affective disorders was not examined. Teens with ADHD displayed smaller BLA volumes (van Dessel et al., 2020) and a relationship was found between peer problems and anxiety in this population (Mikami et al., 2011). Additionally, previous studies examining total amygdala volumes in relation to anxiety symptoms in TD children (6–13 years) have reported that decreased volumes predicted self‐reported anxiety scores (Warnell et al., 2018).

The lateral nucleus of the amygdala, part of the BLA complex, is the major site receiving inputs from visual, auditory, somatosensory, olfactory, and gustatory systems. The lateral nucleus projects to the other nuclei in the BLA including the basal and accessory basal nuclei. Information is then transmitted through the intercalated cells, and to the Ce nucleus, which sends out information through amygdala efferents (Duvarci & Pare, 2014) toward the hypothalamus, the periaqueductal gray, and modulatory systems involved in arousal.

Ce nucleus plays a central role in physiological and behavioral responses to fearful and stressful stimuli. The Ce receives sensory inputs from cortical and subcortical regions and is the major output nucleus of the amygdala and expresses high levels of pro‐ and anti‐stress peptides. In particular, the Ce nucleus expresses corticotropin releasing factor (CRF), which is a neuropeptide that regulates the hypothalamic–pituitary–adrenal axis, the body's stress response system. Regulation of CRF expression in the Ce nucleus is essential for adaptation to chronic stress (Keen‐Rhinehart et al., 2009).

The larger Ce nuclei volumes seen in the current study in children with ASD and OCD, and the association with anxiety behaviors may reflect an imbalance in the stress response system. Genetic and/or environmental factors that contribute to alterations in the size of Ce nucleus may lead to altered CRF expression, and dysregulation of stress responses that may overall influence anxiety behaviors.

The AAA volumes were also associated with higher anxiety scores in children with ASD and OCD. This small cluster of cells in the amygdala complex is a target of the orexinergic system (Sah et al., 2003). Orexins are involved in emotions, hunger and stress and mediate behavioral arousal through projections originating in the hypothalamus that target the amygdala among other regions. The role of the AAA and anxiety remains unexplored and findings in the current study should be examined in larger samples.

Although elevated anxiety levels were present in children and adolescents with ADHD compared to TD children and adolescents, the relationship between anxiety and amygdala subnuclei volumes seen in participants with ASD and OCD was not found in children and adolescents with ADHD. Children and adolescents with ADHD reported less anxiety than children with ASD and OCD thus future research is needed to explore whether the mechanisms underlying anxiety in ASD and OCD differs from anxiety experienced by children and adolescents without these disorders. Anxiety scores were taken from the CBCL anxiety subscale as these scores were accessible from most participants. While the CBCL‐A is a commonly used measure, additional studies could incorporate other anxiety scales with expanded criteria to investigate the prevalence of different types of anxiety in these populations of children and adolescents.

## 7 LIMITATIONS

While all data collection occurred at the same site, there was a scanner upgrade. The upgrade of the scanner did not have any significant impact on the quality of scans obtained nor was there an influence on amygdala volumes. Although a slightly different imaging protocol was implemented following the scanner upgrade, all scans were registered to the same FreeSurfer template, and all were placed within the same anatomical space. As such, the volumes of the amygdala subnuclei were unlikely to be unduly influenced by the imaging protocol. We did not investigate the intelligence quotient (IQ) in our analyses as measures of IQ were not obtained in all individuals due to a lack of resources. Future work could determine the association of amygdala subnuclei volumes, IQ, and diagnoses of ASD, ADHD, OCD, and TD children and adolescents. In our work, we also did not address symptom severity based on standardized assessments in children with neurodevelopmental disorders. Standardized assessments to confirm the primary diagnosis were administered to some children as part of the experimental protocol. Future research examining the association of symptom severity in children with NDDs in relation to anxiety behaviors and amygdala subnuclei development is warranted. Additionally, prolonged anxiety may have downstream effects on brain development through activation of endogenous stress systems. In turn, longitudinal studies are needed to fully characterize the association between anxiety and brain development in NDDs and OCD. We analyzed a large heterogeneous dataset from children and adolescents with NDDs, OCD and TD children and adolescents. We did not have any specific hypotheses related to age, but this would be an important area of future work. Future work should also look toward combining genetic data such as from Enhancing Neuro‐Imaging Genetics through Meta‐Analysis (ENIGMA) of structural brain regions including the amygdala with regards to clinical populations (Thompson et al., 2020). An additional consideration is that we relied on parent reports of anxiety as a measure of children's anxiety through the use of the CBCL‐A. Inclusion of a self‐report measure of anxiety could also be of importance as anxiety is an internalizing disorder and as such, a child's report may more accurately capture their personal experience better than the interpretation of an external rater, such as a parent. A wide range of anxiety scores were reported in the children using the CBCL‐A and only some children and adolescents scored in the clinically significant range. One limitation of this test is the use of shared items between the anxiety and attention behaviors subscales, which may potentially conflate anxiety scores for ADHD children. The use of multiple anxiety assessment tools, including both child and parent reports, may result in the most accurate anxiety measures (Vasa et al., 2016). Additional measures may also better capture atypical manifestations of anxiety, which may present in children with NDD or OCD, but not TD children and adolescents.

## 8 CONCLUSIONS

Although ASD, ADHD, and OCD are regarded as distinct clinical disorders, recent research is increasingly recognizing the overlap of their clinical phenotypes and underlying neurobiology. This work contributes to a growing body of literature examining such overlap. Through segmenting nine subregions of the amygdala, we demonstrated that the volume of the Ce and AAA nuclei predicted anxiety scores. The Ce nucleus volume, the main output nucleus of the amygdala was a key predictor of anxiety in children and adolescents with ASD and OCD. Whether this enlargement is the result of increased anxiety or contributes to increased anxiety requires further investigation. Further study of the role the amygdala nuclei play in both typical and atypical functioning will increase our understanding of how these neural regions contribute to behavioral pathologies, such as anxiety.

## AUTHOR CONTRIBUTIONS

Evdokia Anagnostou, Russell Schachar, Rob Nicolson, Jason P. Lerch, Christopher Hammill, Jennifer Crosbie, Elizabeth Kelley, Muhammad Ayub, Xudong Liu, Jessica Brian, Paul D. Arnold, and Stelios Georgiades were involved in study concept, study design and implementation as well as data acquisition. Diane Seguin, Sara Pac, Jianan Wang, Rob Nicolson, Julio Martinez‐Trujillo, and Emma G. Duerden were involved in developing the research question, developing analytic tools, and analyzing the data. Diane Seguin, Sara Pac and Emma G. Duerden wrote the manuscript. All authors reviewed the manuscript.

## CONFLICT OF INTEREST

The authors declare no competing interests.

## Data Availability

The datasets generated during and/or analysed during the current study are available in the Brain‐Code repository, https://www.braincode.ca/.
